# Indoor air quality in public utility environments—a review

**DOI:** 10.1007/s11356-017-8567-7

**Published:** 2017-02-24

**Authors:** Monika Śmiełowska, Mariusz Marć, Bożena Zabiegała

**Affiliations:** Department of Analytical Chemistry, Faculty of Chemistry, Gdańsk University of Technology, Narutowicza Str. 11/12, PL 80-233 Gdańsk, Poland

**Keywords:** Indoor environment quality, Public health, Indoor pollutants, Public utilities, Human exposure

## Abstract

Indoor air quality has been the object of interest for scientists and specialists from the fields of science such as chemistry, medicine and ventilation system design. This results from a considerable number of potential factors, which may influence the quality of the broadly understood indoor air in a negative way. Poor quality of indoor air in various types of public utility buildings may significantly affect an increase in the incidence of various types of civilisation diseases. This paper presents information about a broad spectrum of chemical compounds that were identified and determined in the indoor environment of various types of public utility rooms such as churches, museums, libraries, temples and hospitals. An analysis of literature data allowed for identification of the most important transport paths of chemical compounds that significantly influence the quality of the indoor environment and thus the comfort of living and the health of persons staying in it.

**Electronic supplementary material:**

The online version of this article (doi:10.1007/s11356-017-8567-7) contains supplementary material, which is available to authorized users.

## Introduction

**Fig. 1:**
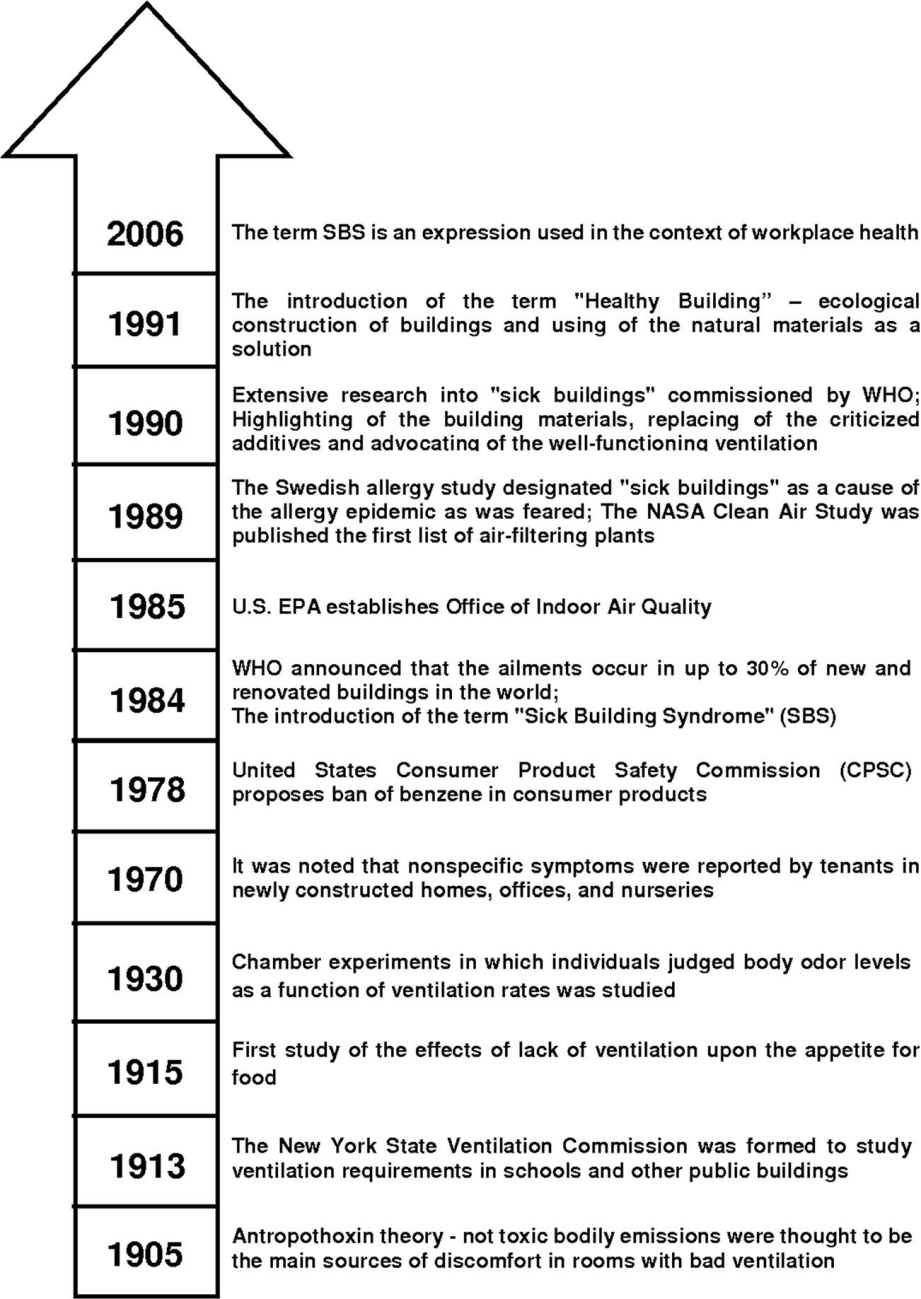
The milestones in extending of knowledge on the indoor air quality (IAQ) control

Until the 1970s, it was considered that the quality of the air in all kinds of enclosed spaces is influenced only by the pollution present in the atmospheric air surrounding this space. Also, in the 1970s, as a result of an energy crisis, the concept of designing and building residential rooms and public utility buildings was changed (Righi et al. 2001). Actions were mostly focused on making new rooms as airtight as possible (e.g. by using PVC windows, using thermal insulation wool, or by insulating building walls with a layer of styrofoam of an appropriate thickness) and on practical elimination of thermal energy between the house and the outdoor environment (Ng et al. 2012). From the economic and energy point of view, such a concept was exactly right. However, an increase in the tightness of buildings and the resulting reduction in air exchange between the residential room and the surrounding environment caused a significant increase in the content of chemical compounds in the indoor environment of confined spaces (Missia et al. 2010). This led to the occurrence of a real threat to human health—also due to the fact that xenobiotics present in the indoor environment can be factors adversely affect the cardiovascular, immune and respiratory systems (Zhang and Smith 2003; Zabiegała 2006; Shinohara et al. 2009). The correlation of the incidence of diseases amongst users of the so-called “airtight” buildings with the level of the content of many pollutants influenced the change in the concept of considering the indoor environment. An increased level of concentrations of selected chemical compounds, as compared to atmospheric air, and the appearance of completely “new” pollutants implied that the indoor environment should be regarded as a separate research area (Takeuchi et al. 2014). Since then, it has been acknowledged that the type and quantity of chemical compounds (both organic and inorganic), which are present in various types of residential rooms and public utility buildings are influenced—apart from ventilation (Lyng et al. 2015)—by two main factors (Hu et al. 2007; Jia et al. 2008; Salthammer and Bahadir 2009; Azuma et al. 2016): (i) the presence and types of decoration and finishing materials including building and structural materials and (ii) all kinds of activities and actions undertaken by users of a given room. The information about the so-called milestones in the development of knowledge on the assessment of the degree of indoor air pollution was presented in Fig. 1 (Jones 1999; Seinfeld 2004; Sundell 2004; Weschler 2009; Persily 2015).

In nearly every publication and scientific study concerning air quality, information can be found that the average adult person spends from 70 to 90% of his/her time indoors (Jones 1999; Delgado-Saborit et al. 2011; Hamidin et al. 2013). The time spent in various types of indoor air by a person is an individual characteristic, and it depends on a different social and environmental factors including (Brasche and Bischof 2005; Salthammer and Bahadir 2009) (i) the users’ age—a much greater part of the day is spent indoors by children, mothers taking care of children and elderly people; (ii) the geographical latitude—in particular, inhabitants of moderate climate zones—amongst other things due to temperatures that do not ensure thermal comfort for a large part of the year, and, as a result, the inhabitants of these zones spend a large part of time indoors; (iii) the type of work—employees sometimes spend at their workplace indoors 8 up to even 12 h, depending on their job; and (iv) lifestyle and quality of life—this factor mostly depends on the way of spending one’s leisure time—whether it is spend actively outdoors or indoors (sports halls, gyms, swimming pools).

Each room intended for permanent or temporary stay should be considered individually as a specific microenvironment with a varying influence of physiochemical factors on the quality of indoor air. At present, specialists’ attention is focused on defining the sources of emissions in the indoor environment (their origin and characteristics), transport paths, the chemical composition of indoor air and the way in which a broad spectrum of chemical compounds occurring in the indoor environment in various amounts influence the human body (Uhde and Salthammer 2007).

The paper presents information derived from the literature overview, which concerns the quality of indoor air in various types of public utility buildings (municipal and university libraries, churches, temples, office rooms, etc.). Information was presented on the main transport paths, and factors that significantly influence the type and quantity of chemical compounds were present in indoor air of public utility environments. In addition, information was also presented on xenobiotics that are usually detected and determined in indoor air and the analytical procedures and measurement techniques used at the stage of detection, identification and quantitative determination of chemical compounds.

## Air quality in various types of public places

Considering the human exposure to air pollution in everyday life, air quality at public utility places is quite important. In this case, the extent of the exposure resulting from the presence of harmful chemical compounds in the air depends on xenobiotic concentrations and the exposure time to them. It results from the available literature data that the average person spends a considerable amount of time in various public utility premises (even up to 40% of a day). Moreover, it can be concluded that selected social groups stay at such places in a non-random and regulated manner; e.g. students at schools and the intensity of their exposure to selected volatile chemical compounds will be different than, for example, during a one-off visit to an office of an adult person. In descriptions of human exposure to harmful substances, two notions are used—instantaneous and chronic exposure (Liang and Liao 2007).

In the context of health effects, the type of volatile chemical compounds to which a person is exposed is also important. The type of air pollutants that are present at public utility places is determined by several factors such as

- The intensity of air exchange (actual values and ones resulting from regulations),
- The specificity of a given place (e.g. the use of specific equipment, substances or undertaking certain characteristic activities which generate emissions of pollutants into the atmosphere),
- Emissions from various types of indoor materials and indoor equipment,
- Temperature and relative humidity in the studied indoor area,
- The ventilation system, purification of the air supplied to the building,
- The presence of external sources of emissions,
- The quality of atmospheric air surrounding a given building,
- The formation of secondary pollutions.

In the research work on the control of the quality of indoor air at public places, the attention is mostly focused on the volatile organic compounds (VOCs). Amongst chemical compounds, considered in the aforementioned research, the following can be distinguished: BTEX, aldehydes, terpenes, organic acids, phthalates, chlorinated hydrocarbons and halogenated organic compounds. On the other hand, a part of research focuses on examining the content of inorganic chemical compounds, which can take part in reactions with VOCs, resulting in the formation of secondary pollutants, which might be more toxic than primary pollution (Morrison and Nazaroff 2002; Wolkoff et al. 2006; Wang and Morrison 2006).

It can be noticed that the primary sources of pollution at various public utility places are similar. They are mostly differentiated by the nature of the studied indoor environment and the purpose for which it is used. This factor influences additional sources of emissions or, for defined sources, changes the extent of the influence of individual sources in the formation of the indoor air composition.

### Air quality in museums

Some gaseous pollutants have a destructive effect on exhibits at the museum, causing their corrosion and decomposition. Pollutants that are a serious hazard include acetic and formic acids, acetic aldehyde, formaldehyde, sulphur compounds and ozone (Supplementary Table 1). These pollutants come from both primary and secondary emissions. For example, ozone, which is the main component of photochemical smog in outdoor air, can be formed as a result of imperfect operation of the air purification system indoors (of course only if the studied indoor areas are equipped with such a system). One of the sources of ozone in indoor air can also be the operation of office equipment (e.g. photocopiers) (Aschmann et al. 2002; Atkinson and Arey 2003; Hubbard et al. 2005).

The influence of the museum location can be seen in the results of the research conducted by Chianese et al. (2012). The authors examined the air quality in a museum situated near an airport, a street with high traffic intensity and a park. In the authors’ opinion, this location has resulted in the presence of the BTEX, naphthalene and benzoic acid (the influence of transport) as well as limonene (the influence of vegetation-biogenic sources) in the indoor air at museums.

Krupińska et al. (2013) paid attention to the fact that pollutants from the outside are transported to the museum area mostly through gaps in the structure of the building, open windows, doors and ventilation systems. Undesirable pollution can be also transported from the outside by museum employees and tourists. It was shown that the levels of the SO_2_ and O_3_ in the air inside the museum were significantly lower than those measured outside the building, whilst NO_2_ concentrations were disproportionately higher or equal to the values measured outside. According to the authors, this may be the effect of a higher reactivity of SO_2_ and O_3_. These gases could have reacted with wood-based materials present at the museum. Such a possibility was also signalled by Chianese et al. (2012), who paid attention to a very low level of O_3_ in the indoor air, which could indicate either a lack of indoor emission sources of this gas or its degradation resulting from reactions with chemical compounds being part of the chemical composition of the exhibits.

Moreover, Krupińska et al. (2013), during one of measuring campaigns, observed the presence of organic acids in the air at the museum (Antwerp, Belgium). This fact was associated with emissions from wooden materials. It was concluded that wooden exhibits should be stored and exhibited at reduced temperatures as emissions of organic compounds from wooden materials increase together with the temperature. The necessity to reduce to the minimum the use of wooden showcases and shelves in museums was also emphasised due to the release of large quantities of VOCs, which are the substrate for secondary pollutants (including carboxylic acids). Also, during research on the indoor air quality in rooms of a museum in Hanover (Germany), attention was paid to an increased quantity of acetic acid, especially at the Department of Ethnology where exhibits that are mostly made of tropical wood are kept. The presence of formic acid in the museum air was explained by the fact that it is used for the tanning of animal hides (Department of Zoology, Department of Prehistory) and also the possibility of the occurrence of secondary chemical reactions of direct formaldehyde oxidation on the surface of materials in an alkaline environment and in the presence of formic acid (Cannizzaro reaction) (Schieweck et al. 2005).

The air quality in museum areas depends on the type of exhibits that are stored in them. Generally, the following types of museums can be distinguished according to the kind of exhibits:

1. Museums of architecture,
2. Archaeological museums,
3. Biographical museums,
4. Museums of medicine and pharmacy,
5. Wax museums,
6. Historical museums,
7. Museums of means of transport,
8. Museums of literature,
9. Museums of craftsmanship,
10. Natural history museums,
11. Sacral museums,
12. Museums of art,
13. Technical museums,
14. Museums of toys,
15. Regional museums.

Research by Schieweck et al. (2005) shows the influence of the specificity of the museum on the air quality inside it. Increased concentrations of formaldehyde were observed at the Zoology and Prehistory Department, which can be explained by the fact that some of the exhibits were preserved in formalin. At the Art Gallery, on the other hand, high formaldehyde content is related to the sources of emissions in the form of a large quantity of wood-based materials. The formaldehyde level does not exceed the admissible concentration (according to German recommendations 120 μg/m^3^) in any of the rooms mentioned above; however, employees of the museum can be exposed to larger amounts of formaldehyde through direct exposure to vapours during the organisation of the exhibition.

The presence of monoterpenes in the indoor air, in the authors’ opinion, results from the presence of furniture with drawers made of soft pine in some of the rooms. BTEX, styrene and benzaldehyde come from emissions from finishing materials, which were used during the renovation of some of the analysed rooms (Schieweck et al. 2005).

### Air quality in libraries

Considering the sources of VOC emissions in indoor environments such as libraries, attention should be paid to the possibility of these compounds being emitted from materials that books are made of paper, parchment, leather, cardboard, etc. It was shown that paper products could be one of the sources of formaldehyde in indoor air. Kim et al. (2013) stated in their studies that formaldehyde released from paper does not affect significantly the air quality in libraries. The authors claim that the air quality in libraries is mostly influenced by the emissions of organic compounds from equipment and finishing materials, i.e. furniture, panels and flooring. Similar conclusions were drawn by Chao and Chan (2001), who indicated that in a Hong Kong-based library (China), where the air quality was studied, the following items or elements were present: suspended ceilings, carpet on the floor and walls painted with acrylic emulsion. It was found that in these rooms, concentrations of VOCs and formaldehydes were much lower than in other public utility room, where similar research was conducted and where the walls were covered with plaster or wallpaper. This might imply that acrylic paint is safe for the finishing of walls in rooms since it is a source of emissions of marginal significance (from the point of view of the emission rate).

The control of pollution of air in libraries is important not only for the health of people but also for keeping the book collection in good condition, especially in museum libraries and in libraries of historical importance. Historical book collections, which are not stored in suitable conditions as regards the temperature, relative humidity and also the air quality, can undergo accelerated degradation. The presence of gases such as NO_2_ and SO_2_ in the air with increased relative humidity lowers the pH value of paper. This causes the decomposition of cellulose fibres in the paper structure, and as a result, the paper turns yellow and brittle. Decomposition of cellulose fibres is also promoted by the presence of ozone—a strong oxidant (Begin et al. 1999; Menart et al. 2014). The problem of the environment of libraries (especially of historical importance) also includes the agents used for the preservation and maintenance of collections, e.g. methylcyclohexane (Hatakeyama and Akimoto 1991; Cincinelli et al. 2016).

Research conducted by Andretta et al. (2016) in a historical library shows that higher NO_2_ concentrations are observed in the summer, just as in the case of O_3_. This can result from differences in the relative humidity in the library during the two seasons. As explained by the authors, the relative humidity was higher in the room during winter than in the summer. A high value of relative humidity promotes the chemical reactions of NO_2_, as a result of which HONO or HNO_3_ are formed. According to the authors, this phenomenon could cause significant changes in NO_2_ concentrations in the air. The influence of the operation of ventilation systems is also important, as their effectiveness may influence the NO_2_ from the atmospheric air to enclosed spaces. The presence of NO_2_ in the atmospheric air is usually conditioned by the presence of vehicle traffic. Undoubtedly, this factor also contributes to the indoor air quality in the analysed library. In the Supplementary Table 2, the information on analytical procedures, which found application during the aforementioned research work, was listed.

### Air quality in temples and churches

Temples are public utility buildings for performing sacred rituals. Several hundred people can be present there. Customs and rituals specific for a given culture are reflected in the quality of the air in the room where they are performed. It can be concluded that the status of the indoor air quality in a temple is considerably influenced by the geographical latitude of the kind of religious building. The diversity of rites and rituals in individual countries results in the use of materials characteristic of a given culture and religion with a specific intended use that can influence the emissions of specific compounds to the gaseous phase (Mleczkowska et al. 2016).

The research on air quality conducted at a church (Szalowa, Poland) by the scientific team of Worobiec et al. (2007) provided information that the level of inorganic pollutants in the air in this village small church is low, and mostly, it is caused only by the transport of gases from the air surrounding the building. Sulphur dioxide was not detected in the indoor air in the mentioned church. The authors suppose that this is caused by a high speed of gas deposition on equipment and subsequent oxidation to sulphates. The majority of Roman Catholic churches have wooden equipment in the form of pews, sculptures, beams, etc. One of factors having a negative effect on wood causing its slow degradation is the presence of ozone in the air. In the atmospheric air, the highest concentration of ozone occurs mostly in cities with increased traffic intensity on sunny days. Ozone is a secondary pollutant and it might get into religious buildings through the ventilation system. This, in turn, influences the quality of indoor air. At high temperatures, typical of summer, the kinetics of ozone reactions with organic compounds from wood increases—this leads to reduced ozone concentrations in indoor air. For this reason, higher ozone concentrations were observed in the church air during winter than in summer. NO_2_ concentrations, on the other hand, both in the winter and in summer, were similar to the concentrations in the atmospheric air surrounding the building. In winter, however, the pollution was two to three times higher than in the summer, which, according to the authors, was caused by increased emissions from external sources—related to energy combustion. It is interesting that NO_2_ concentrations in the altar area were elevated, regardless of the season in which measurements were performed, which was influenced by burning candles in this part of the church.

Burning incense and candles is an indispensable part of many rituals in temples. This, in turn, translates into air quality in temples. Incense, both natural and made from various synthetic substances, emits a lot of pollutants as a result of the burning process (Wang et al. 2007). It was shown that the incensing ritual is a significant source of VOCs, and exposure to compounds emitted during this ritual may be connected to a disadvantageous influence on health (Jetter et al. 2002). The results of research conducted by Zhang et al. (2015) revealed variable concentrations of formaldehyde and BTEX, depending on the burnt incense, which is related to the occurrence of the so-called “rush hours,” during which specific religious rituals take place. The average admissible level of formaldehyde content in some Chinese temples was exceeded several times (values higher than 100 μg/m^3^). The levels of BTEX content exceeded the guidelines proposed by WHO experts. It was shown that the highest values were obtained in the immediate surroundings of vessels used for burning incense and in cult rooms, which confirms that the incensing ritual is the main source of VOC emissions.

It Indian culture, it is a common practice to burn selected natural materials during specific rites. This ritual is often accompanied by sprinkling “oils and holy waters” onto the flames, which changes the nature of the burning process. The highest emissions of VOCs were observed in research conducted by Dewangan et al. (2013) during a wedding ceremony. This is related to the fact that during wedding ceremonies, various natural and synthetic materials are used (e.g. cow dung cakes, cow urine, semi-clarified butter, wood, dry leaves, oils and camphor). During the research, an attempt was made to compare the condition of the air in Hindu and Buddhist temples according to the various types of materials that are burnt during rituals. The emission rates were compared, i.e. concentrations expressed as the mass of pollutants emitted from kilogramme of burned material during rituals in both temples. The results allowed one to conclude that the air in a Hindu temple contains fewer pollutants (Supplementary Table 3). This results from the use of materials such as cotton and vegetable oil during burning rituals, which do not cause high VOC emissions into the air in the temple.

### Air quality in schools

Inadequate air quality in school buildings can cause health problems in students and teachers and also affect the comfort of learning and working. The necessity of ensuring suitable air quality in classrooms, thus promoting better well-being and health of students and school employees, should be a priority. Some schools do not have a mechanical ventilation system; this problem particularly affects schools situated in smaller towns. For this reason, the ventilation is often supplemented by airing rooms (opening doors and windows). In such a case, concentrations of pollutants, apart from emissions from materials of the equipment, largely depend on the concentrations of compounds in the atmospheric air (Stabile et al. 2016). A very important aspect in the field of the indoor air quality monitoring in school buildings is the presence of not only VOCs but also semi-volatile organic compounds (SVOCs) and polychlorinated biphenyls (PCBs). According to literature data, people that spend a most of their time in school buildings (teachers, students and other employees) might have much higher concentration level of PCBs in their body (in plasma or serum) in comparison to non-exposed people (Herrick et al. 2016).

To identify sources of VOCs in the air in school buildings, Madureira et al. (2015) compared the ratios of concentrations of chemical compounds measured both inside and outside buildings (I/O). High values of I/O ratios (I/O > 6) for D-limonene, formaldehyde and acetic aldehyde revealed that indoor sources of emission are responsible for the presence of these compounds, whilst the low value of the I/O ratios for benzene (I/O = 0.84) revealed that the benzene concentration is similar both in the indoor and atmospheric air. This implies that exchange with atmospheric air has the most important influence on the presence of benzene in the analysed school buildings. At the same time, attention was paid to the fact that the highest VOC levels in the air were observed in an art classroom, which is connected with emissions from paints and adhesives, etc., that are used there (Supplementary Table 4). This confirms the significance of the activity of people at school on the quality of indoor air. The determination of numerical values of I/O ratio is widely used in literature studies concerning the quality of indoor environment. Information about the I/O ratio allows to estimate in a very easy and quick way the relationship between indoor and outdoor concentration of defined chemical compound or selected group of chemical compounds. Due to this fact, it is possible to indicate in a simple way potential factors or sources of chemical compounds that might influence on the quality of indoor environment. Higher values of I/O ratio correspond to the fact that the emission source of defined chemical compound or a group of chemical compounds is mainly located in indoor environment (Chen and Zhao 2011; Krupińska et al. 2013; Bari et al. 2015; Xu et al. 2016).

An important issue is the assessment of air after renovation works. An analysis of literature data shows that the highest concentration of the VOCs in indoor air is observed in newly built or renovated rooms. New school buildings that have just been commissioned are a special case. Before starting normal operation of the school, a standard procedure should involve intensive and long-term airing of rooms (seasoning). Fresh finishing and building materials used show a high size of emissions of the VOCs into the indoor environment (Hodgson et al. 2000). Lim-Kyu et al. (2012) noticed this phenomenon in their research conducted in a new school building in Seoul (Korea). Over a year, three measuring campaigns were conducted to monitor changes in the VOC content in the air. On their basis, it was concluded that to minimise exposure to elevated VOC values, it was necessary to season the building (by heating rooms and intensive ventilation) for at least 6 months to allow the concentrations of harmful compounds to fall below the admissible limit. Such a procedure minimises the phenomenon of the so-called sick building syndrome (SBS), whose symptoms include headaches, fatigue and fainting in rooms with poor quality of indoor air (Joshi 2008).

### The quality of air in offices

Offices and agencies are a certain microenvironment with specific air quality. Mostly, office employees stay in such rooms (on a regulated basis) as well as customers (on a non-regulated basis). Apart from ventilation, the air quality in offices is mostly influenced by the type of equipment and the finishing materials used. For this reason, a significant element is ensuring optimal exchange of air resulting from broadly understood comfort, taking into account the functions of these rooms, the heat and humidity balance and the presence of solid and gaseous pollutants in the air.

Considering the results obtained from the research on air quality in offices and agencies, which are presented in Supplementary Table 5, it can be concluded that BTEX is one of the most frequently occurring air pollutants in offices. The majority of aromatic hydrocarbons may come from both emissions from equipment materials and from migration from atmospheric air, where vehicle transport is the source of emissions (Kim et al. 2001; Chao and Chan 2001).

Compounds that were found in offices include chlorinated hydrocarbons and halogenated organic compounds. The sources of emissions of these compounds into indoor air are all washing agents, carpets, adhesives, etc. Dichloromethane and chloroform are commonly used as solvents in the process of production of printer housings, some furniture and cushions. Dichloromethane is often used as a solvent in many production processes of products made of plastic materials. Halogenated organic compounds, on the other hand, are used as insecticides (Chao and Chan 2001).

If office employees complain of symptoms connected with poor air quality, a possible solution that reduces the VOC content in the air is the application of special ozone-generating devices, which react with VOCs and cause their degradation. Ozone-generating units are equipped with total volatile organic compound (TVOC) sensors, which are used to modulate ozone release. For this reason, ozone concentrations in the air when the ozonation process does not occur are very low. Literature data show that the VOC content in an office room equipped with such devices can be reduced by nearly a half (Srivastava and Devotta 2007). The control of the level of pollutants that are formed on a secondary basis remains a problem.

### Air quality in hospitals

The basic task of health care centres involves ensuring medical assistance and nursing care to patients. There are various facilities of this type and also various departments, which specialise in a specific scope of medical services, e.g. operating theatres, intensive care units and radiology departments. The diversity resulting from the functions of various facilities and rooms determined the nature and quantity of VOCs in indoor air. Moreover, three groups of persons, which can be found in a hospital, can be distinguished, which are patients, employees and visitors. Differences in the health in each of these groups and the diversity of devices that are found in hospitals make the hospital microclimate more complex, and it differs from the environment in other public utility buildings. Air quality control in hospital rooms in terms of biological (bacterial and mycotoxins) and chemical hazards plays an important role in infection prevention in hospitals—it is aimed at protecting both personnel and patients, especially ones with reduced or impaired immunity. Infants and pregnant women are a particularly vulnerable group of patients. Improper control of indoor air quality in hospitals may cause hospital infections and occupational diseases. From the point of view of problems discussed in this paper, the object of interest is the quality of hospital air in terms of the content of organic and inorganic compounds and not biological pollution that is a separate issue (Verde et al. 2015; Gniadek et al. 2011).

Another problem is the presence of radon in the hospital’s environment. Radon is a naturally present radioactive gaseous decay product of uranium. It occurs widely in the environment, especially in rocks and soils with varying concentration (depending on geographical location) and in building materials manufactured from these (Groves-Kirkby et al. 2016). In addition, radon is sometimes used in hospitals (as a special health programme) to treat cancer by the enhancement of the immune system and tumour suppression genes such as p53 (Zdrojewicz and Strzelczyk 2006) and also to treat other diseases (radon baths to prevent auto-immune diseases such as arthritis, endocrine disorders) (Neda et al. 2008). On the other hand, the epidemiological studies have shown a clear relationship between breathing high concentrations of radon and incidence of lung cancer (Field et al. 2002). Due to this fact, radon has to be considered as a significant xenobiotic that might affect the indoor air quality in such specific microenvironment like hospital buildings.

Anaesthetic gases are gaseous pollutants characteristic of hospital environment, which are used as additions to anaesthetics. Gases that are commonly used are halothane, isoflurane, sevoflurane and N_2_O. Modern systems extracting these gases ensure low occupational exposure and negligible release into the environment; however, in poorer countries, older devices and installations are still used, which do not always meet safety parameters. The results of the research conducted at hospitals (Athens, Greece) show that the parameters defining levels of anaesthetic substances in the air of operating theatres include anaesthetic equipment, use of a scavenging system, operations of the mechanical ventilation system, anaesthesia procedure followed during sampling, time distance from previous anaesthesia procedure and change of anaesthetic gas container. Chronic exposure to such pollutants may cause severe damage to the liver and kidneys; it is also harmful to pregnant women as it may cause miscarriages and congenital defects of the foetus (Dascalaki et al. 2008).

Another example of specialist gases which may occur in hospital air are disinfection gases used for cold sterilisation of surgical instruments and utensils. Such gases include formaldehyde, glutaraldehyde and ethylene oxide. Each of these substances is highly toxic if it occurs at high concentrations, and a longer exposure causes asthma, dyspnea, chest pains and irritations. Ethylene oxide and formaldehyde, which occur in the air at high concentrations, have carcinogenic and mutagenic properties (Zeiger et al. 2005; Duong et al. 2011).

The occurrence of the BTEX compounds in hospital rooms is connected with their entry together with atmospheric air as a result of the operation of ventilation systems (Dascalaki et al. 2008; Kheirmand et al. 2014). This explains a high content of BTEX and carbonyl compounds at hospitals in Guangzhou, China, where the poor quality of atmospheric air is observed (Lu et al. 2006). In a hospital in Yazd Province, India, BTEX emissions from electronic devices (printers, copiers, computers) that are used in hospital rooms with other functions (hospital administration) were also considered (Kheirmand et al. 2014). Acetaldehyde, on the other hand, can occur in the air as one of products of metabolic changes (see Supplementary Table 6) (Lu et al. 2006).

A very important problem of the hospital air is also the presence of compounds so-called endocrine disruptors that influence reproduction. These compounds include phthalates, which are commonly used as plastifiers. A lot of equipment, which are present at hospitals, are made of polymer materials (e.g. plastic infusion bags, blood bags, plastic film, injectors and rubber tubing). They can be a potential source of phthalate emissions into the air. The results of research conducted by Wang et al. (2015) showed that the highest phthalate concentrations in hospital air occur in hospital pharmacies and next in transfusion rooms and in hallways. The presence of these compounds in the indoor air was connected with the characteristics of the equipment of rooms and the type of workstations. Phthalates can be emitted from some medical products, which is confirmed by their high content in pharmacies. In the research, attention was also paid to the necessity of increased control of the phthalate content at obstetric wards as the exposure of infants to these compounds causes severe disorders in sexual development during puberty (Meeker and Ferguson 2014).

### Air quality in elderly care centres

Elderly care centres are a specific environment as they are a place of residence for people who stay there, a place of work for the personnel and a place of temporary stay during visits for families of residents. This fact translates into diversification of time, which various groups of people spend at care centres. Residents and patients of care centres are the most exposed to potential pollutants when a large group of people is present on a small surface area. The extent of exposure is augmented by the fact that an older body is more susceptible to negative effects of air pollution. Impaired mobility of elderly people causes increased exposure to pollution occurring in rooms (Mendes et al. 2015).

It was shown that the presence of nitrogen oxides and formaldehyde in the air may cause dyspnea, cough and wheezing, and it can be related to higher probability of the occurrence of chronic respiratory disease. Elderly people are more susceptible to these symptoms. Hence, rooms are frequently aired by care centre employees (Simoni et al. 2003).

The results obtained during research on air quality in care centres in Europe on the VOC content were listed in the Supplementary Table 7. On their basis, seasonal differences in VOC concentrations can be found. Higher VOC concentrations were generally observed in winter. It was found that the TVOC value during the heating season could be even 150% higher in some care centre rooms as compared with values obtained for the summer season (Mendes et al. 2015). This is caused by heating that increases VOC emissions—from the point of view of thermal comfort of users, higher temperatures and lower air exchange intensity increase the VOC level. In the air in rooms where the access of fresh air is limited, chemical compounds can accumulate, which can cause harmful health effects in the human body. These compounds are emitted mostly by heaters, furniture, building materials, etc. (Buczyńska et al. 2014). Another additional source of emissions is the need to use disinfectants—after spraying such agents into the air, terpenes can be formed, amongst other things, which are a common ingredient of the majority of disinfectants (Coleman et al. 2008).

Concentrations of indoor air pollutants are influenced not only by emissions from the equipment but also by the specificity of behaviour of people (activity) in such rooms. This phenomenon was found in the research described in numerous papers (Walgraeve et al. 2011; Mendes et al. 2015). The average person spends approximately one third of the day in the bedroom. In care centres, however, this time depends on the state of health of residents. Completely dependent persons are classified as bedridden patients, often with rooms with other bedridden patients. Their activity is limited to a minimum. Independent persons have rooms where they spend a part of their daily life. The activity of physically fitter persons may contribute to an increase in emissions of air pollutants in rooms where these persons stay. Activities that generate VOCs include activities connected with care, preparation and consumption of small meals and entertainment (Mui et al. 2008).

## Summary

The quality of indoor air in public utility buildings in which people can stay during the day is influenced by a range of various environmental factors. Generally, they can be divided into three main groups:

(i) Factors resulting from all kinds of human activity in a given indoor environment;
(ii) Factors conditioned by specific characteristics of a given indoor area;
(iii) The type and quantity of chemical compounds present in atmospheric air surrounding the indoor environment.

The analysis of literature data on indoor air quality in various public utility premises leads to the conclusion that regardless of the place or region where research is conducted, the problem with the occurrence of elevated concentrations of chemical compounds in indoor air is still valid and remains unsolved in many regions. Due to broad diversity and specificity of public utility buildings, the qualitative and quantitative compositions of indoor air can undergo dynamic changes. For this reason, no uniform and precise legal regulations have been developed, yet concerning the quality of indoor air that would take into account information about the type and admissible levels of the content of chemical compounds in public utility buildings. In many cases, the results of research on indoor air quality are compared with information contained in law regulations or guidelines, which defines the type and quantity of chemical compound, which can occur in atmospheric air. Such comparisons only make it possible to obtain informative or cognitive data, which is not measurable in terms of the value of the information on the quality of indoor air.

Problems connected with defining and creating appropriate regulations and guidelines on indoor air quality in various types of public utility environments result from the fact that some of these indoor areas are defined as a place of work, e.g. offices, schools, museums and hospitals. The problem consists of defining, by using appropriate legal regulations, what the work environment is in a precise manner and what environmental conditions should be met there and what parameters should be monitored in them on a routine basis. For this reason, there exists the urgent need for social consultations and routine monitoring research on indoor air quality in various types of public utility buildings to create a suitable database. Collecting such information on the type and quantity of chemical compounds in various indoor environments in which people can stay will make it possible to take legislative action to draw up appropriate legal regulations aimed at improving the quality of indoor air.

It is important not only to create the regulations on the quality of indoor air but also to define tools, techniques and methods, which make it possible to define the content of chemical compounds in an indoor environment. One should also take into account the necessity of establishing appropriate entities, which would monitor indoor air quality on a routine bases and help to solve problems effectively if the concentrations of defined chemical compounds in indoor air exceed the highest admissible values. For this reason, one should not only devote special attention to the improvement of the quality of atmospheric air in urban areas but also increase efforts and administrative actions to improve air quality in public utility buildings.

Work should be started from scratch by choosing a suitable location for an enclosed space, designing an appropriate ventilation system and filters and choosing suitable construction and structural elements as well as equipment and finishing materials. All of these activities should also take into account the intended use of the enclosed space, the frequency of its use, the number of users and potential pollutants which can be present in indoor air and have a distinct influence on users’ health and comfort.

## Electronic supplementary material

Supplementary Table 1 Analytical procedures used in the study of air quality in the European and Asian libraries. (DOC 41 kb)

Supplementary Table 2 Analytical procedures used in the study of air quality in the European elderly care centres. (DOC 30 kb)

Supplementary Table 3 Analytical procedures used in the study of air quality in the European museums. (DOC 36 kb)

Supplementary Table 4 Analytical procedures used in the study of air quality in the European and Asian temples. (DOC 33 kb)

Supplementary Table 5 Analytical procedures used in the study of air quality in the European and Asian schools. (DOC 33 kb)

Supplementary Table 6 Analytical procedures used in the study of air quality in the European and Asian hospitals. (DOC 39 kb)

Supplementary Table 7 Analytical procedures used in the study of air quality in the European and Asian offices. (DOC 37 kb)
