# Supplementary material for: Indoor air quality in public utility environments—a review
Source: Environ Sci Pollut Res Int. 2017 Feb 24;24(12):11166–76. doi: 10.1007/s11356-017-8567-7 (PMC5393278; doi:10.1007/s11356-017-8567-7)
Supplement: Supplementary file 1 — Analytical procedures used in the study of air quality in the European and Asian libraries. (DOC 41 kb) [file 11356_2017_8567_MOESM1_ESM.doc]

| **Localization**  **Supplementary Table 1**. Analytical procedures used in the study of air quality in the European museums. | **Determined compounds** | **Sampling technique** | **Used sorbent** | **Technique of separation/liberation analytes** | **Final determination technique** | **Concentration** | **Determination of PM10 and PM2,5** | **Ref** |
| --- | --- | --- | --- | --- | --- | --- | --- | --- |
| Museum, Antwerp, Belgium | SO2, NO2, formic acid, acetic acid | Passive (Radiello samplers) | Microporous polyethylene tube coated with triethanolamine (TEA) | Extraction with water | IC | 2008-2009:  NO2 21.7 µg/m3  SO2  0.4 µg/m3  2009-2010:  NO2 18.6 µg/m3  SO2 0.8 µg/m3  2011-2012:  NO2 25.0 µg/m3  SO2 < LOD  Formic acid 36.0 µg/m3  Acetic acid 144.5 µg/m3 | Sampling using Teflon membrane filters (Pallflex) and gravimetric analysis | (Krupińska et al. 2013) |
|  | O3 | Passive (Radiello samplers) | Microporous polyethylene tube filled with silica gel coated with 4,4’-dipyridylethylene | Condensation with MBTH | UV/VIS-spectrophotometry | 2008-2009: 1.5 µg/m3  2009-2010: 2.0 µg/m3  2011-2012: 1.5 µg/m3 |  |  |
| Museum, Naples, Italy | BTEX, benzoic acid, naphthalene and limonene | Passive – during 1 month (Radiello samplers) | Activated charcoal | Thermal desorption | GC-MS | Benzene 5.7 µg/m3  Toluene 14.1 µg/m3  Ethylobenzene 2.4 µg/m3  Xylenes 17.9 µg/m3  Benzoic acid 1.0 µg/m3  Naphthalene 0.7 µg/m3  Limonene < LOD | Sampling using Dust Scan Scout, Aerosol monitor | (Chianese et al. 2012) |
|  | O3, NOx­, SO2, HNO2, | Passive – during 1 month (Analyst samplers) | --- | Extraction with solution mady by NaHCO3 (1mM) and Na2CO3 (3,2 mM) with the addition of 0,03 % H2O2 | IC | O3 0.9 µg/m3  NOx 3.7 µg/m3  SO2 < 0.5 µg/m3  HNO2 0.3 µg/m3 |  |  |
| Museum, Hanover, Germany | Formaldehyde | Dynamic – air flow rate 2 l/min; during 40 min | Distilled water | --- | Derivatization and photometrically determination | Zoology Dep. 29.5 µg/m3  Ethnology Dep.<LOD  Prehistory Dep. 54.0 µg/m3  Art gallery 63.0 µg/m3 | --- | (Schieweck et al. 2005) |
|  | Organic acids | Dynamic – air flow rate 2 l/min; during 60 min | Solution 0,1 N NaOH | --- | IEC | Formic acid:  Zoology Dep. 29.0 µg/m3  Ethnology Dep. 32.0 µg/m3  Prehistory Dep. 90.8 µg/m3  Art gallery <10 µg/m3  Acetic acid:  Zoology Dep. <1.3 µg/m3  Ethnology Dep. 195.3 µg/m3  Prehistory Dep. <5.0 µg/m3  Art gallery 59.0 µg/m3 |  |  |
|  | VOCs | Dynamic – air flow rate 0,15 l/min; during 40 min | Tenax TA | Thermal desorption | GC-MS | Toluene:  Geology Dep. 6.5 µg/m3  Zoology Dep. 8.0 µg/m3  Ethnology Dep. 11.7 µg/m3  Prehistory Dep. 6.0 µg/m3  Art gallery 21.0 µg/m3  Xylenes:  Geology Dep. <LOD  Zoology Dep. 0.5 µg/m3  Ethnology Dep. 3.3 µg/m3  Prehistory Dep.<LOD  Art gallery 38.0 µg/m3  Styrene:  Geology Dep. 7.0 µg/m3  Zoology Dep. 2.0 µg/m3  Ethnology Dep. 6.3 µg/m3  Prehistory Dep. <LOD  Art gallery 26.0 µg/m3  α-pinene  Geology Dep. 114.5 µg/m3  Zoology Dep. 4.5 µg/m3  Ethnology Dep. 16.3 µg/m3  Prehistory Dep.<LOD  Art gallery 35.0 µg/m3  3-carene  Geology Dep. 54.0 µg/m3  Zoology Dep. <LOD  Ethnology Dep. 5.7 µg/m3  Prehistory Dep. <LOD  Art gallery 16.0 µg/m3  Benzaldehyde:  Geology Dep. 6.5 µg/m3  Zoology Dep. 32.0 µg/m3  Ethnology Dep. 15.0 µg/m3  Prehistory Dep. 18.5 µg/m3  Art gallery 17.0 µg/m3  TVOC  Geology Dep. 226.0 µg/m3  Zoology Dep. 104.0 µg/m3  Ethnology Dep. 386.7 µg/m3  Prehistory Dep. 370.5 µg/m3  Art gallery 2101.0 µg/m3 |  |  |
