# Supplementary material for: Indoor air quality in public utility environments—a review
Source: Environ Sci Pollut Res Int. 2017 Feb 24;24(12):11166–76. doi: 10.1007/s11356-017-8567-7 (PMC5393278; doi:10.1007/s11356-017-8567-7)
Supplement: Supplementary file 2 — Analytical procedures used in the study of air quality in the European elderly care centres. (DOC 30 kb) [file 11356_2017_8567_MOESM2_ESM.doc]

| **Localization**  **Supplementary Table 2**. Analytical procedures used in the study of air quality in the European and Asian libraries. | **Determined compounds** | **Sampling technique** | **Used sorbent** | **Technique of separation/liberation analytes** | **Final determination technique** | **Concentration** | **Determination of PM_10_ and PM_2,5_** | **Ref** |
| --- | --- | --- | --- | --- | --- | --- | --- | --- |
| Libraries, Seoul, Korea | Aldehydes | Dynamic – air flow rate 0,5 l/min; during 30 min | 2,4-DNPH | Extraction with acetonitrile | HPLC-UV/VIS | Public library:  Formaldehyde 29.2 µg/m^3^  Acetaldehyde 8.9 µg/m^3^  Propionaldehyde 4.4 µg/m^3^  Benzaldehyde 3.9 µg/m^3^  Hexaldehyde 4.9 µg/m^3^  Children library:  Formaldehyde 29.3 µg/m^3^  Acetaldehyde 7.2 µg/m^3^  Propionaldehyde 3.3 µg/m^3^  Benzaldehyde 2.4 µg/m^3^  Hexaldehyde 4.9 µg/m^3^ | --- | (Kim et al. 2013) |
| Historical Library, Ravenna, Italy | O_3_ | Passive – during 1 week (Palmes tubes, Passam Company) | Filter soaked with a solution of 1,2-di(4-pyridyl)ethylene (DPE) | --- | Spectrophotometric | Summer 25.0 µg/m^3^  Winter 4.8 µg/m^3^ | --- | (Andretta et al. 2016) |
|  | NO_2_ | Passive – during 2 weeks (Palmes tubes, Passam Company) | Filter impregnated with trietanoloamina (TEA) | --- | Adding N-(1-naphthyl)ethylenediamine dihydrochloride (NEDA) and spectrophotometric | Summer 8.0 µg/m^3^  Winter 2.0 µg/m^3^ |  |  |
| Library, Hong Kong, China | VOCs | Stainless steel canister- collection during 4 h | --- | concentration by collecting in a cryogenically-cooled trap and thermal desorption | GC-MS | Aromatic hydrocarbon 40.0 µg/m^3^  Chlorinated hydrocarbon 30.0 µg/m^3^  Organohalogen 10.0 µg/m^3^ | --- | (Chao and Chan 2001) |
