# Supplementary material for: Indoor air quality in public utility environments—a review
Source: Environ Sci Pollut Res Int. 2017 Feb 24;24(12):11166–76. doi: 10.1007/s11356-017-8567-7 (PMC5393278; doi:10.1007/s11356-017-8567-7)
Supplement: Supplementary file 3 — Analytical procedures used in the study of air quality in the European museums. (DOC 36 kb) [file 11356_2017_8567_MOESM3_ESM.doc]

| **Localization**  **Supplementary Table 3**. Analytical procedures used in the study of air quality in the European and Asian temples. | **Determined compounds** | **Sampling technique** | **Used sorbent** | **Technique of separation/liberation analytes** | **Final determination technique** | **Concentration** | **Determination of PM_10_ and PM_2,5_** | **Ref** |
| --- | --- | --- | --- | --- | --- | --- | --- | --- |
| Church, Szalowa, Poland | SO_2_, NO_2_ | Passive – during 5 days (Radiello samplers) | Microporous polyethylene tube coated with triethanolamine (TEA) | Extraction with water | IC | Near the altar  WINTER  SO_2_ <LOD  NO_2_ 10.0 µg/m^3^  SUMMER  SO_2_ <LOD  NO_2_ 3.0 µg/m^3^  Near the organ  WINTER  SO_2_ <LOD  NO_2_ 5.0 µg/m^3^  SUMMER  SO_2_ <LOD  NO_2_ 3.0 µg/m^3^ | Using Nucleopore filters and gravimetric analysis | (Worobiec et al. 2007) |
|  | O_3_ | Passive – during 5 days (Radiello samplers) | Microporous polyethylene tube filled with silica gel coated with 4,4’-dipyridylethylene | Condensation with MTBH | UV/VIS-spectrophotometry | Near the altar  WINTER 14.0 µg/m^3^  SUMMER 5.0 µg/m^3^  Near the organ  WINTER 12.0 µg/m^3^  SUMMER 5.0 µg/m^3^ |  |  |
| Temple 1, Shanxi Province, China | VOCs | Dynamic – air flow rate 0.5 l/min | --- | Thermal desorption | GC-MS | Benzene 21.0 µg/m^3^  Toluene 30.0 µg/m^3^  Xylene 40.0 µg/m^3^  Formaldehyde 150.0 µg/m^3^ | Using particle counter Fluke 983 | (Zhang et al. 2015) |
| Temple 2, Shanxi Province, China |  |  |  |  |  | Benzene 22.5 µg/m^3^  Toluene 22.5 µg/m^3^  Xylene 32.0 µg/m^3^  Formaldehyde 110.0 µg/m^3^ |  |  |
| Temple 3, Beijing, China |  |  |  |  |  | Benzene 92.0 µg/m^3^  Toluene 102.8 µg/m^3^  Xylene 91.3 µg/m^3^  Fomaldehyde 515.0 µg/m^3^ |  |  |
| Buddhist temple, Raipur, India | VOCs | Passive (Radiello samplers) | Carbograph 4 | Thermal desorption | GC-MS | Benzene 0.893 g/kg  Toluene 0.570 g/kg  Ethylbenzene 0.081 g/kg  Xylenes 0.291 g/kg  Styrene 0.790 g/kg | Using quartz  fiber filters and gravimetric analysis | (Dewangan et  al. 2013) |
| Hindu temple, Raipur, India |  |  |  |  |  | Benzene 0.011 g/kg  Toluene 0.006 g/kg  Ethylbenzene 0.002 g/kg  Xylenes 0.010 g/kg  Styrene 0.0090 g/kg |  |  |
