# Supplementary material for: Indoor air quality in public utility environments—a review
Source: Environ Sci Pollut Res Int. 2017 Feb 24;24(12):11166–76. doi: 10.1007/s11356-017-8567-7 (PMC5393278; doi:10.1007/s11356-017-8567-7)
Supplement: Supplementary file 4 — Analytical procedures used in the study of air quality in the European and Asian temples. (DOC 33 kb) [file 11356_2017_8567_MOESM4_ESM.doc]

| **Localization**  **Supplementary Table 4**. Analytical procedures used in the study of air quality in the European and Asian schools. | **Determined compounds** | **Sampling technique** | **Used sorbent** | **Technique of separation/liberation analytes** | **Final determination technique** | **Concentration** | **Determination of PM_10_ and PM_2.5_** | **Ref** |
| --- | --- | --- | --- | --- | --- | --- | --- | --- |
| 20 primary schools, Porto, Portugal | VOCs | Passive - during 5 days | Tenax TA | Thermal desorption | GC-MS | Benzene 2.5 µg/m^3^  Toluene 6.4 µg/m^3^  m/p-xylene 5.0 µg/m^3^  o-xylene 2.3 µg/m^3^  d-limonene 23.1 µg/m^3^  α-pinene 1.8 µg/m^3^  Trichloroethylene <LOD  Tetrachloroethylene 2.9 µg/m^3^  Naphthalene 1.3 µg/m^3^  Styrene 1.2 µg/m^3^  TVOCs 140.3 µg/m^3^ | Using TSI DustTrak DRX photometers | (Madureira et al. 2015) |
|  | Aldehydes | Passive - during 5 days (Radiello samplers) | 2,4-dinitrophenylhydrazine (2,4-DNPH) coated Florisil | Extraction with acetonitrile | HPLC-UV/VIS | Formaldehyde 17.5 µg/m^3^  Acetaldehyde 7.7 µg/m^3^ |  |  |
| New school building; Seoul, Korea | VOCs | Passive – during 3 weeks | Charcoal | Extraction with CS_2_ | GC-FID | March 2010  Benzene 10.7 µg/m^3^  Toluene 1372.1 µg/m^3^  Ethylbenzene 48.5 µg/m^3^  Xylene 94.9 µg/m^3^  Styrene 5.5 µg/m^3^  α-pinene 26.7 µg/m^3^  Limonene 6.8 µg/m^3^  TVOCs 1704.4 µg/m^3^  September 2010  Benzene 1.4 µg/m^3^  Toluene 83.4 µg/m^3^  Ethylbenzene 12.5 µg/m^3^  Xylene 15.1 µg/m^3^  Styrene 1.8 µg/m^3^  α-pinene 2.6 µg/m^3^  Limonene 1.0 µg/m^3^  TVOCs 192.5 µg/m^3^  February 2011  Benzene 25 µg/m^3^  Toluene 46.5 µg/m^3^  Ethylbenzene 9.2 µg/m^3^  Xylene 20.1 µg/m^3^  Styrene 1.7 µg/m^3^  α-pinene 1.8 µg/m^3^  Limonene 5.7 µg/m^3^  TVOCs 101.9 µg/m^3^ | --- | (Lim-Kyu et al. 2012) |
