# Supplementary material for: Indoor air quality in public utility environments—a review
Source: Environ Sci Pollut Res Int. 2017 Feb 24;24(12):11166–76. doi: 10.1007/s11356-017-8567-7 (PMC5393278; doi:10.1007/s11356-017-8567-7)
Supplement: Supplementary file 5 — Analytical procedures used in the study of air quality in the European and Asian schools. (DOC 33 kb) [file 11356_2017_8567_MOESM5_ESM.doc]

| **Localization**  **Supplementary Table 5**. Analytical procedures used in the study of air quality in the European and Asian offices. | **Determined compounds** | **Sampling technique** | **Used sorbent** | **Technique of separation/liberation analytes** | **Final determination technique** | **Concentration** | **Determination of PM_10_ and PM_2,5_** | **Ref** |
| --- | --- | --- | --- | --- | --- | --- | --- | --- |
| Office, Birmingham, United Kingdom | VOCs | Dynamic – air flow rate 40 ml/min; during 2 h | Carbopack B followed by Carbosieve SIII | Thermal desorption | GC-MS | 1,3-butadiene 0.3 µg/m^3^ | --- | (Kim et al. 2001) |
|  |  |  | Tenax GR followed by Carbotrap |  |  | Benzene 5.9 µg/m^3^  Toluene 22.0 µg/m^3^  Ethylbenzene 2.4 µg/m^3^  o-xylene 1.8 µg/m^3^  m-xylene 6.0 µg/m^3^  p-xylene 1.7 µg/m^3^  Styrene 0.6 µg/m^3^  Naphthalene 1.7 µg/m^3^  1,4-dichlorobenzene 0.1 µg/m^3^ |  |  |
| Office, Mumbai, India | VOCs | Dynamic – air flow rate 20 ml/min (personal air sampler Staplex PST 3000 A) | Chromosorb 106 | Thermal desorption | GC-MS | Benzene 44.92 µg/m^3^  Toluene 0.82 µg/m^3^  Ethylbenzene 0.06 µg/m^3^  p-xylene 0.03 µg/m^3^  trichloroethylene 0.08 µg/m^3^  Methylene chloride 1251.67 µg/m^3^  Carbon tetrachloride 57.50 µg/m^3^  Chloroform 24.17 µg/m^3^ | --- | (A.Srivastava et al. 2007) |
|  | O_3_ | Dynamic – air flow rate 1 l/min; during 8 h | Alkaline potassium iodide solution | --- | UV-VIS analysis | With ozonization 1.20 ∙ 10^-5^ µg/m^3^  Wihout ozonization 3.14 ∙ 10^-6^ µg/m^3^ |  |  |
| Offices, Hong Kong, China | VOCs | Stainless steel canister- collection during 4 h | --- | Concentration by collecting in a cryogenically-cooled trap and thermal desorption | GC-MS | Aromatic hydrocarbon 121.8 µg/m^3^  Chlorinated hydrocarbon 156.0 µg/m^3^  Organohalogen 46.2 µg/m^3^ | --- | (Chao and Chan 2001) |
