# Supplementary material for: Indoor air quality in public utility environments—a review
Source: Environ Sci Pollut Res Int. 2017 Feb 24;24(12):11166–76. doi: 10.1007/s11356-017-8567-7 (PMC5393278; doi:10.1007/s11356-017-8567-7)
Supplement: Supplementary file 6 — Analytical procedures used in the study of air quality in the European and Asian hospitals. (DOC 39 kb) [file 11356_2017_8567_MOESM6_ESM.doc]

| **Localization**  **Supplementary Table 6**. Analytical procedures used in the study of air quality in the European and Asian hospitals. | **Determined compounds** | **Sampling technique** | **Used sorbent** | **Technique of separation/liberation analytes** | **Final determination technique** | **Concentration** | **Determination of PM_10_ and PM_2,5_** | **Ref** |
| --- | --- | --- | --- | --- | --- | --- | --- | --- |
| 10 Hospitals, Athens, Greece | VOCs | Dynamic | Tenax TA | Thermal desorption | GC-MS | Anaesthetic compounds 2.362 mg/m^3^  Aromatic compounds 0.239 mg/m^3^  Formaldehyde and glutaraldehyde 0.495mg/m^3^  Other aldehydes, oxides, alcohols 1.920 mg/m^3^  Other 3.846 mg/m^3^  TVOC 8.862 mg/m^3^ | --- | (Dascalaki et al. 2008) |
| 4 hospitals, Guangzhou, China | VOCs | Stainless steel canister | --- | concentration by collecting in a cryogenically-cooled trap and thermal desorption | GC-MS | Hospital 1:  Benzene 5.0 µg/m^3^  Toluene 100.0 µg/m^3^  Ethylbenzene 67.5 µg/m^3^  p-xylene 35.0 µg/m^3^  o-xylene 35.0 µg/m^3^  TVOCs 242.5 µg/m^3^  Hospital 2:  Benzene 22.8 µg/m^3^  Toluene 43.8 µg/m^3^  Ethylbenzene 17.5 µg/m^3^  p-xylene 14.0 µg/m^3^  o-xylene 8.8 µg/m^3^  TVOCs 119.0 µg/m^3^  Hospital 3:  Benzene 5.3 µg/m^3^  Toluene 9.0 µg/m^3^  Ethylbenzene 2.0 µg/m^3^  p-xylene 3.3 µg/m^3^  o-xylene 1.0 µg/m^3^  TVOCs 20.8 µg/m^3^  Hospital 4:  Benzene 13.0 µg/m^3^  Toluene 19.0 µg/m^3^  Ethylbenzene 15.0 µg/m^3^  p-xylene 26.0 µg/m^3^  o-xylene 8.0 µg/m^3^  TVOCs 85.0 µg/m^3^ | --- | (Lu et al. 2006) |
|  | Carbonyl compounds | Dynamic – air flow rate 0,8-1,3 l/min; during 2-3 h | Sep-Pak Silica Gel Cartridge (Waters, Millipore Corp.) | Extraction with acetonitrile | HPLC-UV/VIS | Hospital 1:  Formaldehyde 9.8 µg/m^3^  Acetaldehyde 13.7 µg/m^3^  Acetone 45.0 µg/m^3^  Benzaldehyde 2.7 µg/m^3^  Hexanaldehyde 2.3 µg/m^3^  Propionaldehyde 3.4 µg/m^3^  Total 119.0 µg/m^3^  Hospital 2  Formaldehyde 7.4 µg/m^3^  Acetaldehyde 17.7 µg/m^3^  Acetone 28.8 µg/m^3^  Benzaldehyde 2.6 µg/m^3^  Hexanaldehyde 2.9 µg/m^3^  Propionaldehyde 4.8 µg/m^3^  Total 99.5 µg/m^3^  Hospital 3  Formaldehyde 9.8 µg/m^3^  Acetaldehyde 11.4 µg/m^3^  Acetone 19.4 µg/m^3^  Benzaldehyde 1.6 µg/m^3^  Hexanaldehyde 2.2 µg/m^3^  Propionaldehyde 2.1 µg/m^3^  Total 63.7 µg/m^3^  Hospital 4  Formaldehyde 5.7 µg/m^3^  Acetaldehyde 9.9 µg/m^3^  Acetone 18.6 µg/m^3^  Benzaldehyde 2.4 µg/m^3^  Hexanaldehyde 1.5 µg/m^3^  Propionaldehyde 2.4 µg/m^3^  Total 59.5 µg/m^3^ |  |  |
| 5 hospitals, China | Phthalate esters | Dynamic – air flow rate 1 l/min; during 8-10 h | Glass fiber filter and XAD-2 | Extraction with mixture of DCM:acetone 1:1 (v/v) and using ultrasounds | GC-MS | Dimethyl phthalate 1.7 µg/m^3^  Diethyl phthalate 2.6 µg/m^3^  Dibutyl phthalate 3.6 µg/m^3^  Butylbenzyl phthalate 1.9 µg/m^3^  Di(2-ethylhexyl) phthalate 3.1 µg/m^3^  Di-n-octyl phthalate < LOD  Total 13.2 µg/m^3^ | --- | (Wang et al. 2015) |
| 8 hospitals . Yazd province, India | BTEX | Dynamic – using Tedlar bag; during 8 min | PDMS/CAR SPME fiber | SPME fiber desorption | GC-FID | Hospital 1  Benzene 0.31 µg/m^3^  Toluene 0.55 µg/m^3^  Ethylbenzene 0.81 µg/m^3^  Xylene 0.77 µg/m^3^  Hospital 2  Benzene 2.70 µg/m^3^  Toluene 1.45 µg/m^3^  Ethylbenzene 3.19 µg/m^3^  Xylene 0.98 µg/m^3^  Hospital 3  Benzene 2.30 µg/m^3^  Toluene 0.70 µg/m^3^  Ethylbenzene 1.11 µg/m^3^  Xylene 0.60 µg/m^3^  Hospital 4  Benzene 0.5 µg/m^3^  Toluene 0.42 µg/m^3^  Ethylbenzene 0.22 µg/m^3^  Xylene 0.62 µg/m^3^  Hospital 5  Benzene 1.40 µg/m^3^  Toluene 0.48 µg/m^3^  Ethylbenzene 0.30 µg/m^3^  Xylene 0.87 µg/m^3^  Hospital 6  Benzene 2.81 µg/m^3^  Toluene 0.24 µg/m^3^  Ethylbenzene 0.11 µg/m^3^  Xylene 1.31 µg/m^3^  Hospital 7  Benzene 1.76 µg/m^3^  Toluene 3.82 µg/m^3^  Ethylbenzene 0.41 µg/m^3^  Xylene 1.60 µg/m^3^  Hospital 8  Benzene 1.50 µg/m^3^  Toluene 0.39 µg/m^3^  Ethylbenzene 0.15 µg/m^3^  Xylene 0.52 µg/m^3^ | --- | (Kheirmand et al. 2014) |
