# Supplementary material for: Indoor air quality in public utility environments—a review
Source: Environ Sci Pollut Res Int. 2017 Feb 24;24(12):11166–76. doi: 10.1007/s11356-017-8567-7 (PMC5393278; doi:10.1007/s11356-017-8567-7)
Supplement: Supplementary file 7 — Analytical procedures used in the study of air quality in the European and Asian offices. (DOC 37 kb) [file 11356_2017_8567_MOESM7_ESM.doc]

| **Localization**  **Supplementary Table 7**. Analytical procedures used in the study of air quality in the European elderly care centers. | **Determined compounds** | **Sampling technique** | **Used sorbent** | **Technique of separation/liberation analytes** | **Final determination technique** | **Concentration** | **Determination of PM_10_ and PM_2,5_** | **Ref** |
| --- | --- | --- | --- | --- | --- | --- | --- | --- |
| Elderly care center, Porto, Portugal | TVOCs | Dynamic - air flow rate 0,05 l/min; during 45 min | Tenax TA | Thermal desorption | GC-FID | SUMMER:  Dining room 100.0 µg/m^3^  Drawing room 130.0 µg/m^3^  Bedroom 120.0 µg/m^3^  Bedridden 40.0 µg/m^3^  Medical office 60.0 µg/m^3^  WINTER:  Dining room 140.0 µg/m^3^  Drawing room 150.0 µg/m^3^  Bedroom 130.0 µg/m^3^  Bedridden 90.0 µg/m^3^  Medical office 150.0 µg/m^3^ | Sampling using PTFE filters and gravimetric analysis | (Mendes et al. 2015) |
|  | formaldehyde | Dynamic - air flow rate 0,08 l/min | 2,4-DNPH - coated glass fiber filters |  | HPLC | SUMMER:  Dining room < 42.0 µg/m^3^  Drawing room < 42.0 µg/m^3^  Bedroom < 42.0 µg/m^3^  Bedridden < 42.0 µg/m^3^  Medical office < 42.0 µg/m^3^  WINTER:  Dining room < 42.0 µg/m^3^  Drawing room < 42.0 µg/m^3^  Bedroom < 42.0 µg/m^3^  Bedridden < 42.0 µg/m^3^  Medical office < 42.0 µg/m^3^ |  |  |
| Elderly care center, Antwerp, Belgium | TVOCs | Passive - during 6-9 days) | Tenax TA | Thermal desorption | GC-MS | JUNE 2007  Inhabitant room 1: 129.0 µg/m^3^  Inhabitant room 2: 92.0 µg/m^3^  Inhabitant room 3: 46.0 µg/m^3^  Inhabitant room 4: 71.0 µg/m^3^  Recreational room: 40.0 µg/m^3^ | --- | (Walgraeve et al. 2011) |
| Elderly care center, Broechem, Belgium |  |  |  |  |  | FEBRUARY 2008  Inhabitant room 1: 83.0 µg/m^3^  Inhabitant room 2: 108.0 µg/m^3^  Inhabitant room 3: 163.0 µg/m^3^  Inhabitant room 4: 242.0 µg/m^3^  Recreational room: 165.0 µg/m^3^  OCTOBER 2009  Inhabitant room 1: 35.0 µg/m^3^  Inhabitant room 2: 60.0 µg/m^3^  Inhabitant room 3: 121.0 µg/m^3^  Inhabitant room 4: 50.0 µg/m^3^  Recreational room: 35.0 µg/m^3^ |  |  |
| Elderly care center, Borsbeek, Belgium |  |  |  |  |  | SEPTEMBER 2008  Inhabitant room 1: 54.0 µg/m^3^  Inhabitant room 2: 38.0 µg/m^3^  Inhabitant room 3: 142.0 µg/m^3^  Inhabitant room 4: 35.0 µg/m^3^  Recreational room: 90.0 µg/m^3^ |  |  |
| Elderly care center, Hove, Belgium |  |  |  |  |  | FERBRUARY 2009  Inhabitant room 1: 308.0 µg/m^3^  Inhabitant room 2: 300.0 µg/m^3^  Inhabitant room 3: 204.0 µg/m^3^  Inhabitant room 4: 56.0 µg/m^3^  Recreational room: 42.0 µg/m^3^ |  |  |
| Elderly care center, Bonheiden, Belgium |  |  |  |  |  | JUNE 2009  Inhabitant room 1: 35.0 µg/m^3^  Inhabitant room 2: 38.0 µg/m^3^  Inhabitant room 3: 88.0 µg/m^3^  Inhabitant room 4: 83.0 µg/m^3^  Recreational room: 13.0 µg/m^3^ |  |  |
